# Natural Products and Nanotechnology Against Coronavirus Disease 2019

**DOI:** 10.3389/fchem.2022.819969

**Published:** 2022-02-10

**Authors:** Ning Zeng, Xue Chen, Zeming Liu

**Affiliations:** Department of Plastic and Cosmetic Surgery, Tongji Hospital, Tongji Medical College, Huazhong University of Science and Technology, Wuhan, China

**Keywords:** COVID-19, nanotechnology, natural products, kinetic properties, bioavailability

## Abstract

Coronavirus disease 2019 (COVID-19) is a new and severe infectious disease and new global disaster and is spreading rapidly worldwide. Natural products have a long history and have been widely used to treat various acute, chronic, and even life-threatening diseases worldwide. However, the natural products have reduced bioavailability and availability as they have poor kinetic properties, such as large molecular weight, inability to cross lipid membranes, and weak absorption ability. With the rapid development of nanotechnology, using novel nanotechnology in conjunction with natural products can effectively eliminate the molecular restriction of the entry of nanoproducts into the body and can be used to diagnose and treat various diseases, including COVID-19, bringing new strategies and directions for medicine. This article reviews the role and implementation of natural products against COVID-19 based on nanotechnology.

## Introduction

It has been longer than 2 years since the coronavirus disease 2019 (COVID-19) outbreak, which continues to affect human social and economic life in more than 200 countries and regions around the world (Li et al., 2020; Abu-Raddad et al., 2021; Chen B. et al., 2021; Berwanger, 2021). There have been more than 246 million diagnosed cases and 5 million deaths due to COVID-19 worldwide until October 31, 2021, and thousands continue to die daily. The most affected countries include the United States and India, where more than 1.1 million people have died from COVID-19 (Sinha et al., 2021).

The development of vaccines and plasma therapy has impacted the prevention and treatment of COVID-19 profoundly (Benner et al., 2020; Li C. et al., 2021; Tang P. et al., 2021; Barda et al., 2021; Kunze et al., 2021; Macchia et al., 2021; Tartof et al., 2021; Tedder and Semple, 2021); however, a shortage of medical capacity remains in all countries. The application of traditional medicines including nano-loaded natural products, has also been listed as a major treatment strategy to aid the recovery of patients with COVID-19 and combat the global pandemic (Sinha et al., 2021). Our purpose of this review focus on using novel nanotechnology in conjunction with natural products that used to diagnose and treat COVID-19.

## Natural Products

Natural products have a long history and are widely used around the world (Devi et al., 2010; Al-Zahrani et al., 2021; Kaur et al., 2021). At present, nearly 200,000 natural compounds are extracted for medicinal purposes from higher plants, animals, fungi, and marine organisms, mostly from medicinal and aromatic plants (Atanasov et al., 2015; Ahmed, 2016).

Currently, different natural products reportedly exert anti-cancer, anti-oxidation, anti-malarial, anti-anxiety, and anti-organ effects, and treat various cardiovascular diseases effectively, and facilitate the effective treatment of various cardiovascular diseases (Weng et al., 2019; Li L. et al., 2021). Researchers have found that herbs can directly inhibit pathogens associated with common diseases that infect the respiratory tract or coordinate the activities of the immune system to prevent or alleviate infection (Yan et al., 2021). The application and research of natural products has undergone a long development process from the initial drug compound formulation to the isolation and extraction of highly effective compounds with therapeutic potential from plants and animals (Devi et al., 2010). Even during the COVID-19 pandemic, scholars worldwide found an effective drug to prevent and treat COVID-19. Mohammad et al. tried to prevent COVID-19 by studying the pharmacological effects of black cumin seeds and extracted their bioactive compounds (Islam et al., 2021), Salim et al. conducted a molecular link-based study to explore nigellidine and α-hederin in N. sativa compounds as novel severe acute respiratory syndrome coronavirus 2 (SARS-COV-2) inhibitors (Duru et al., 2021). In addition, several high-quality peer-reviewed herbal clinical trials are ongoing (Jotz et al., 2020; Singh et al., 2021).

**FIGURE 1:**
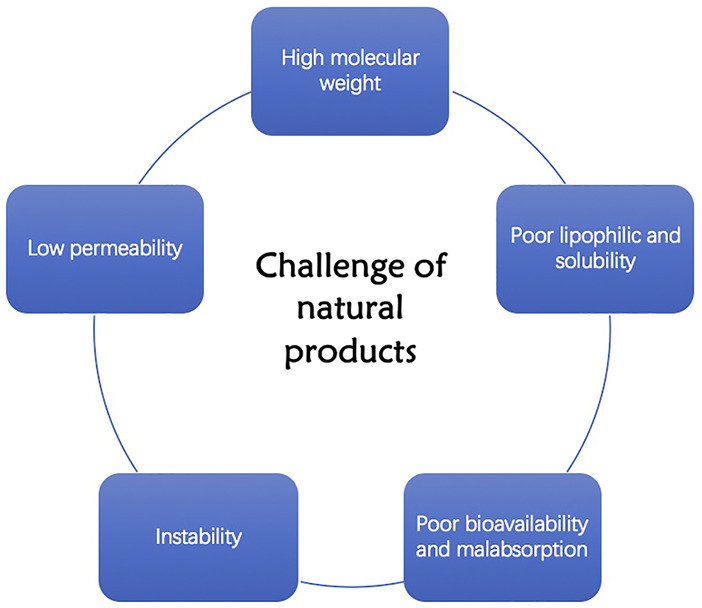
Challenges of natural products.

Compared with synthetic drugs, natural products are easy to obtain, exert several pharmacological effects on different diseases, and their side effects are relatively weak. However, natural products exhibit poor kinetic performance due to characteristics such as their large molecular weight, inability to cross lipid membranes, and weak absorption capacity, which result in reduced bioavailability (Figure 1).

Up to now, there are several natural products (polyphenols, alkaloids, terpenoids etc.) used in COVID 2019 treatment and prevention. Mhatre et al. investigated two tea polyphenols: epigallocatechin-3-gallate and theaflavins and found that they exhibited antiviral activities against positive-sense single-stranded RNA viruses (Mhatre et al., 2021). Typically alkaloids carry one or more nitrogen atom within a heterocyclic ring. In Huang’s study, berbamine (one of alkaloids) was found to inhibit genome replication, and reduced infectious virus production using Vero E6 cells (He et al., 2021). In Pratibha Mishra’s description, Terpenoids also have potential to be developed as a treatment for COVID-19 (Mishra et al., 2021).

## Natural Products Based on Nanotechnology

"Nano" refers to a billionth of a meter in length. Nanomaterials can be manipulated, controlled and modified physically or chemically to acquire specific functional properties. Nanostructures are derived from microscopic structure, changes in size can bring variations in properties, which also makes the application of nanotechnology have great potential (Bustos Cruz et al., 2017; Bayda et al., 2019).

Nanotechnology has developed rapidly in recent years, and natural products, including herbal therapy based on nanotechnology, are becoming increasingly popular and are showing very broad application prospects (Liu et al., 2021; Zhang et al., 2021; Zhu et al., 2021). Nanotechnology usually refers to the use of nanomaterials with sizes between 1 and 100 nm, which can be classified as organic and inorganic nanoparticles. Nanoparticles can be designed in different shapes and sizes, loaded with drugs, and modified functionally and physiochemically according to the characteristics of the active substances. Chemical functionalization and identification of unique physical and chemical properties have been applied in various fields based on the synthesis and characterization of rich engineering materials (Zhang and Tang, 2016; Weiss et al., 2020; Ahmed et al., 2021; Manikandan et al., 2021; Venturi, 2021).

**FIGURE 2:**
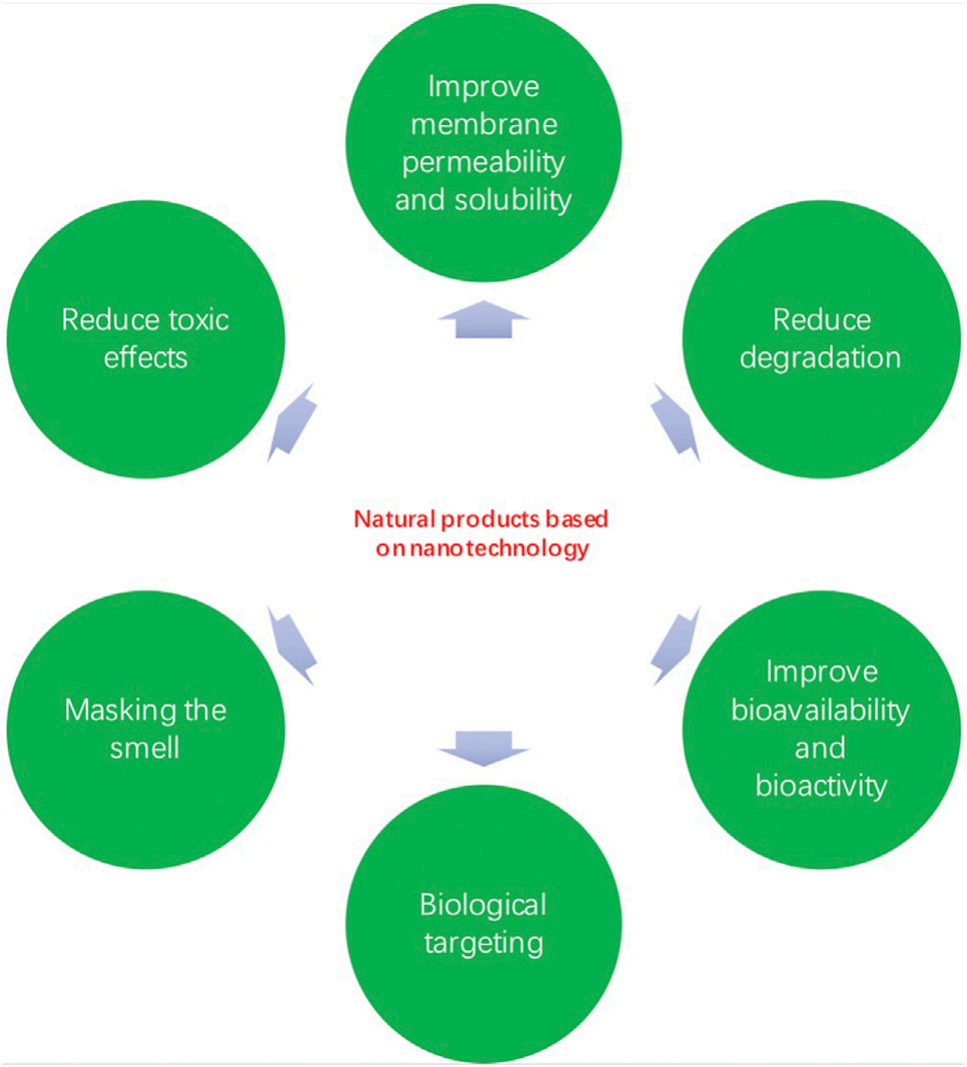
Natural products based on nanotechnology.

**FIGURE 3:**
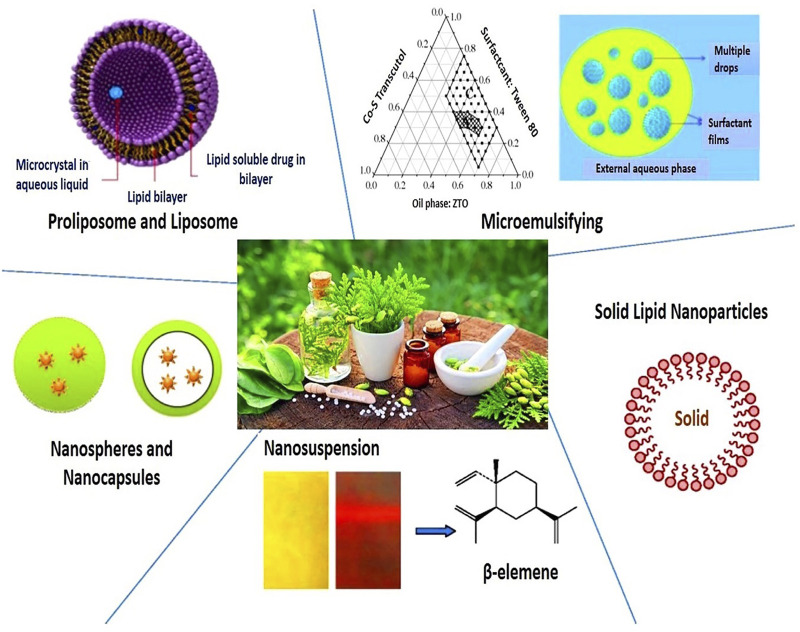
Nanoparticle drug delivery systems for herbal drug formulations (Ahmed et al., 2021).

The amalgamation of novel drug delivery nanocarriers with drug complexes can effectively eliminate the molecular limitations of their entry into the body (Nabi et al., 2019). By using nanocarriers such as nanoparticles, nanoliposomes, and alcohol solutes, natural products can be loaded and smoothly inserted into the appropriate parts of the body (Allaw et al., 2020; Raja et al., 2021; Salarbashi et al., 2021). On the one hand, nanotechnology increases the solubility of a low soluble compound and improves its stability, however, nanotechnology can be combined to enhance the effectiveness of the therapeutic drug and reduce its side effects (Kaur et al., 2021) (Figures 2 and 3). To date, natural products based on nanotechnology have been used to diagnose and treat various diseases, bringing new strategies and directions to medicine (Alfuraydi et al., 2019; Valsalam et al., 2019; Hashemi et al., 2021).

## Natural Products for COVID-19 Treatment Based on Nanotechnology

Diagnostic and therapeutic techniques based on nanotechnology, such as nutritional nanotechnology, have been a concern for researchers since the start of the COVID-19 pandemic, or the development of highly sensitive and specific antigens for COVID-19 detection tests (Layqah and Eissa, 2019). Palmieri et al. conjugated the COVID-19 anti-S protein antibody to a graphene sheet as a sensitive area, preventing antigenic cross-reactivity with MERS-CoV, and successfully detected the virus in clinical samples with high sensitivity and no sample pretreatment (Palmieri and Papi, 2020; Dacrory, 2021). Ahmed et al. developed Au NP-quantum dot nanocomposites that could contain viral lipid tails and promote envelope aggregation and rupture. Layqah et al. also used an Au NP-modified carbon electrode recombinant spike protein SI as a biomarker (Layqah and Eissa, 2019; Lobo-Galo et al., 2021).

The most common therapeutic strategies for resuing existing drugs to treat COVID-19 to reduce severe symptoms in infected patients have worked well, including those previously used safely as herbal therapy medicine, even if they were not originally intended as antivirals (Lobo-Galo et al., 2021). Some of the most promising herbal therapy drugs reduce viral load, length of hospital stay, disease severity, and mortality, and some have been tested in clinical trials (Riva et al., 2020; Tang W.-F. et al., 2021; Hashemi et al., 2021; Jia et al., 2021).

Chloroquine is a typical drug that is extracted from the original long-term application of natural products (Fitch, 1969; Mossaad et al., 2015), and is used to treat malaria after the identification and purification of active components, structural characterization, and understanding of the mechanism of drug action, as well as derivative modification of drugs. During the COVID-19 pandemic, the use of chloroquine to treat COVID-19 was initiated by the State Council of China. In a preliminary *in vitro* study, some trials conducted in patients reported reduced recovery times (Chen Z. et al., 2021; Jia et al., 2021; Roy et al., 2022). The Food Drug Administration approved chloroquine and hydroxychloroquine for emergency use in late March. The main mechanism of action is that entering lung cells can be activated by TMPRSS-2 (Lobo-Galo et al., 2021; Yao et al., 2021). Although some concerns regarding chloroquine remain due to the side effects caused by the drug dosage, the subsequent optimization of loading based on nanotechnology will likely result in improved functionality and efficacy of chloroquine or chloroquine in the treatment of COVID-19 [Table 1(Borba et al., 2020; Duvignaud et al., 2020; Group et al., 2020; Liu et al., 2020; Panda et al., 2020; Tirupakuzhi Vijayaraghavan et al., 2020; Weehuizen and Hoepelman, 2020; Abd-Elsalam et al., 2021; Gautret et al., 2021; Rea-Neto et al., 2021; Thakar et al., 2021)].

**TABLE 1:** Main clinical trials of Chloroquine for Covid-19.

	Author	Design	Country	End point	References
1	Philippe Gautret et al.	Open-label non-randomized clinical trial	France	Viral load	Gautret et al. (2021)
2	RECOVERY Collaborative Group et al.	Randomized, controlled, open-label platform trial	United Kingdom	Mortality	Group et al. (2020)
3	Duvignaud A. et al.	Phase III, multi-arm (5) and multi-stage (MAMS), randomized, open-label controlled superiority trial	France	Prevent hospitalisation or death	Duvignaud et al. (2020)
4	Prasan Kumar Panda et al.	Open label, Parallel arm design, stratified randomised controlled trial	India	Severe category	Panda et al. (2020)
5	Xi Liu et al.	Prospective, open-label, randomized controlled, multicenter clinical study	China	Clinical recovery time	Liu et al. (2020)
6	Mayla Gabriela Silva Borba et al.	Parallel, double-masked, randomized, phase IIb clinical trial	Brazil	Safety and lethality outcomes	Borba et al. (2020)
7	Bharath Kumar Tirupakuzhi Vijayaraghavan et al.	Multi-centre open-label parallel group randomized controlled trial	India et al.	The proportion of healthcare workers developing laboratory confirmed COVID-19 infection within 6 months of randomization	Tirupakuzhi Vijayaraghavan et al. (2020)
8	Alok Thakar et al.	Randomized clinical trial	India	Clinical progression and outcomes	Thakar et al. (2021)
9	Sherief Abd-Elsalam et al.	Randomized clinical trial	Egypt	Recovery within 28 days, the need for mechanical ventilation, and death	Abd-Elsalam et al. (2021)
10	Álvaro Réa-Neto et al.	Randomized, open-label, controlled, phase III trial	Brazil	Clinical status measured on day 14	Rea-Neto et al. (2021)
11	Weehuizen JM et al.	Controlled, open label, cluster-randomized, superiority trial	Netherlands	Disease progression	Weehuizen and Hoepelman (2020)

Active substances extracted and purified from various herbs have long been used as antiviral drugs. These active substances are usually designed based on natural composite structures. Homohar-ringtonine, isolated from Cephalotaxus, is another classic example of the use of herbal compounds against herpes viruses (Hassan, 2020). In addition, natural drug research strategies against COVID-19 include not only studies of one or more biomolecules *in vivo*, but also those conducted in silico derived from natural products with therapeutic or preventive potential to guide and assist in the successful treatment of COVID-19 (Ang et al., 2020).

Curcumin, a polyphenol extracted from the rhizome of turmeric (Kocaadam and Sanlier, 2017), is one of the most thoroughly studied molecules derived from dietary natural products. Its potential benign effects and safety of curcumin on inflammation, cancer, depression, and many other diseases in particular have been investigated (Gao et al., 2004; Hurley et al., 2013; Minassi et al., 2013; Thimmulappa et al., 2021). The related mechanisms include curcumin’s viral inhibition, regulation of inflammation, and immune response. Recently, Zahedipour et al. also preliminarily investigated curcumin’s potential to reverse brosis-associated pulmonary edema and pathways in COVID-19 infection and demonstrated its potential pharmacodynamic potential in the treatment of COVID-19 (Zahedipour et al., 2020). However, curcumin’s biopharmaceutical limitations and limitations on biological responses include physicochemical properties that reduce its bioavailability (Sanidad et al., 2019). Douglas et al. highlighted nanotechnology as a key way to overcome curcumin drug deficiencies and exert anti-COVID-19 efficacy (Dourado et al., 2021). Researchers have developed nanotechnological carriers such as nanoemulsions, liposomes, and nanogels to load curcumin, enhance its solubility, protect it from chemical and metabolic degradation, and modify it to be more easily transported through biofilms for its efficacy. In the current COVID-19 pandemic, there are three curcumin-based nanotechnology products available on the market in the form of polymer nanoparticles (NanocurcTM), liposomes (LipocurcTM), and nanoparticles (sinacurcu-min ^®^), which show initial benign therapeutic effects (Valizadeh et al., 2020; Saber-Moghaddam et al., 2021; Tahmasebi et al., 2021).

Particles with different materials and structures are appear in different ways, such as lipid-based nanoparticles, polymer nanoparticles, and inorganic nanoparticles (Zazo et al., 2016). In addition to the advantages of nanoparticles in transporting large drug molecules, they also have the advantage of being easy to customize and functionalize (Gagliardi, 2017). The mechanism of action of nanoparticles to exert an anti-viral effect is realized through several pathways, such as 1) virus inactivation (direct or indirect), 2) virus action in host cells, 3) virus penetration, and 4) virus replication, depending on the nature and functionalization of the nanoparticles used (Chen and Liang, 2020). In this way, nanoparticles can physically or chemically block these steps and modify the structure of capsid proteins, thereby reducing the viral load (Gurunathan et al., 2020).

## Conclusions and Perspectives

COVID-19 poses a huge challenge to and seriously threatens human health and economic development. In this context, many natural products have attracted great attention as promising anti-SARS-COV-2 drugs and have shown some degree of effectiveness. The present review focuses on recent progress in the application of some important natural products. However, some natural products may have disadvantages and limitations, such as poor water solubility and low bioavailability, which restrict their widespread clinical application. Therefore, there is still a long way to go before drug discovery and development based on natural products can be achieved.

In contrast, nanomaterials have unique physical and chemical properties, it has inherent resistance to pathogenicity and/or its ability to produce inactivated viruses through photothermal catalytic induction of reactive oxygen species (ROS). Secondly, nanoloading natural products can enhance the biocompatibility of natural products and minimize toxicity and side effects, thereby overcoming many obstacles such as adverse reactions. Nano-drug treatment strategies may therefore be a more effective therapeutic approach. In addition, the concept of "nano immunity" can help us design immunomodulatory materials, and has good prospects for COVID-19 development or against cytokine storm (Chauhan et al., 2020). The current study on the etiology of novel coronavirus and the pharmacological mechanism of natural products, in addition to the mature application of nano-drug delivery technology and the improvement of clinical trials of drugs such as chloroquine, natural products based on nanotechnology will provide a broad application prospect for the treatment of COVID-19.

## Abbreviations

COVID-19, coronavirus disease; SARS-CoV-2, severe acute respiratory syndrome coronavirus 2; MERS-CoV, middle east respiratory syndrome coronavirus; TMPRSS-2, transmembrane serine protease 2.
